# A Novel Digital Twin Architecture with Similarity-Based Hybrid Modeling for Supporting Dependable Disaster Management Systems

**DOI:** 10.3390/s22134774

**Published:** 2022-06-24

**Authors:** Seong-Jin Yun, Jin-Woo Kwon, Won-Tae Kim

**Affiliations:** Future Convergence Engineering Major, Department of Computer Science and Engineering, Korea University of Technology and Education, Cheonan 31253, Korea; hiysr0308@koreatech.ac.kr (S.-J.Y.); qweddww@koreatech.ac.kr (J.-W.K.)

**Keywords:** digital twin, hybrid modeling, disaster spread simulation, disaster prediction, wildfire spread simulation

## Abstract

Disaster management systems require accurate disaster monitoring and prediction services to reduce damages caused by natural disasters. Digital twins of natural environments can provide the services for the systems with physics-based and data-driven disaster models. However, the digital twins might generate erroneous disaster prediction due to the impracticability of defining high-fidelity physics-based models for complex natural disaster behavior and the dependency of data-driven models on the training dataset. This causes disaster management systems to inappropriately use disaster response resources, including medical personnel, rescue equipment and relief supplies, to ensure that it may increase the damages from the natural disasters. This study proposes a digital twin architecture to provide accurate disaster prediction services with a similarity-based hybrid modeling scheme. The hybrid modeling scheme creates a hybrid disaster model that compensates for the errors of physics-based prediction results with a data-driven error correction model to enhance the prediction accuracy. The similarity-based hybrid modeling scheme reduces errors from the data dependency of the hybrid model by constructing a training dataset using similarity assessments between the target disaster and the historical disasters. Evaluations in wildfire scenarios show that the digital twin decreases prediction errors by approximately 50% compared with those of the existing schemes.

## 1. Introduction

The need for dependable disaster management systems is increasing as climate changes due to global warming resulting in more hazardous natural disasters such as wildfires, typhoons, and flooding than in previous decades [1]. For example, in the case of a wildfire, the scale of burned lands for the last decade has increased by 15% compared with those of the previous decade [2]. Disaster management systems prevent or mitigate damages after disasters by efficiently organizing disaster response resources, including medical personnel, rescue equipment, relief supplies and maintenance facilities. Since resources are limited, improper organization of the resources cannot mitigate the damages and might lead to worse results than other responses [3]. Therefore, the disaster management system should be operated based on an accurate analysis about where a disaster occurs and how it spreads to deploy the right resources at the right time and place.

Digital twins can provide disaster monitoring and spread prediction services for disaster management systems to support the efficient operation of the systems. Digital twins are virtual replicas of physical systems and natural environments [4]; they provide the services for the system with various computing features, including IoT, AI, big data, and simulation with physics-based and data-driven disaster models. The monitoring and prediction services are the most important features for effective disaster response of the disaster management systems [5]. Therefore, it is necessary to use models that can precisely simulate the natural disasters for the digital twin to provide accurate services.

The physics-based models for the digital twin are implemented with explicit expressions based on natural science about the disasters [6]. The physics-based models might have prediction errors because it is impractical to define a high-fidelity model with explicit expressions for complex and dynamic natural disaster behaviors, including fuel model representation, and dynamic weather changes [7]. Data-driven models are designed without explicit expression by extracting disaster behaviors from observation data using machine learning algorithms [8]. With the disaster behavior model, the data-driven model can perform more accurate disaster spread prediction than the physical-based model. However, the model can cause unexpected errors in the untrained disaster conditions because the data-driven models depend on the training data. The errors by the models of the digital twins can accumulate during prediction. This makes it difficult for the disaster management systems to effectively respond to the disaster.

Recently, hybrid modeling schemes have been proposed to develop a high-fidelity hybrid model by fusing the physics-based model and the data-driven model [9]. The hybrid model can make high-accuracy predictions by complementing each other’s limitations. The physics-based model of the hybrid model prevents unexpected prediction errors from the data-driven model by providing stable reference prediction results. The insufficient fidelity of the physics-based model is supplemented by the data-driven model. In disaster prediction services, the data-driven models can compensate for the error caused by the inaccurate expression of the physics-based models for the disaster behaviors. However, the accuracy of the hybrid model might decrease in harsh disaster conditions where the digital twins have difficulties in acquiring observation data for training the hybrid model.

In this study, we propose a digital twin software architecture with similarity-based hybrid modeling. The hybrid modeling scheme creates a hybrid disaster model by converging a physics-based disaster spread model with a data-driven error correction model. The digital twin can provide accurate disaster spread prediction services using the hybrid model by compensating for prediction errors of the physics-based model with the data-driven model. The similarity-based hybrid modeling technique trains the model with dataset by similarity assessments of the target disaster with the historical disasters. This technique enables the digital twin to find historical data with similar features to the disaster, and to utilize the historical data as training data for the high precision hybrid model. The stored observation data of the historical disasters are used to train a hybrid model for the target disaster. In addition, a model update procedure using the observation data from the target disaster is adopted to continuously improve the prediction performance.

The remainder of this study is organized as follows. In Section 2, we review the existing research about the disaster digital twin. Section 3 describes the proposed digital twin architecture. Section 4 evaluates the digital twin. Section 5 concludes this study.

## 2. Related Works

### 2.1. Digital Twin Software Architecture

In the existing research, the digital twin has been defined in various ways, from a representation model for the physical system and natural environments to an independent computing process that provides optimization services for the physical systems with various ICT technologies [10].

In the early stages of digital twin technologies, digital twins were defined as only a model for physical systems, such as visualization, design, and analysis models. Schleich defined a digital twin with a design model for a physical system [11]. He devised a method to use IoT data in the design model as an input for the system development stage. Boschert defined the digital twin as a sophisticated simulation model [12]; a sophisticated simulation technique for performing state prediction was presented by synchronizing a simulation model for a vehicle with IoT data collected from a running vehicle. In these approaches, each digital twin was defined as a model used in application systems. Since digital twins are strongly coupled with application service logic, it is difficult to utilize digital twins in various applications.

Recently, the existing research defined digital twins as independent computing processes that can be utilized in various domains and applications. Zhao proposed a digital twin architecture combined with artificial intelligence technology to ensure the accurate control of the micro punching machine [13]. The digital twin analyzes operation data with a machine learning model and a simulation model to improve the operation accuracy of the machine. Steindl defined the digital twin software structure based on the microservices architecture [14]. The microservice-based digital twin has functional scalability for computing features for digital twin services, such as data acquisition, data storage, state prediction, and fault analysis. In these approaches, application service developers should design digital twins for performance. This makes it difficult to utilize the digital twins in various application systems because application service developers need to understand both ICT technologies and domain knowledge for the digital twins and the application systems.

### 2.2. Modeling Scheme for the Disaster Digital Twin

Table 1 shows the four implementation types of the natural disaster model and their characteristics for the digital twin.

The physics-based models are implemented with explicit expressions of natural phenomena based on a theory defined by human observation. They are actively used in various disaster spread simulations. Finney developed a fire growth simulator called FARSITE based on Rothaermel’s model [15]. Real disaster growth is so complex and dynamic that it is impractical to define a high-fidelity physics-based model that ensures accurate prediction of disaster progress.

The existing research on physics-based models with data assimilation schemes is conducted to enhance the prediction accuracy of physics-based models. Srivas proposed an ensemble Kalman filter-based wildfire spread prediction scheme [7]. The technique corrected the probabilistic factors of the prediction results of the physics-based model by using the observation data to ensure that high-accuracy prediction can be achieved. Yen proposed a rainfall simulation scheme for typhoons based on the ensemble Kalman filter [16]. They showed that accurate rainfall prediction was possible by correcting the translation speed of typhoons using the ensemble Kalman filter. However, it was difficult to apply it to predict the state of the disaster in progress because it was impossible to obtain observation data for future natural disaster growth.

Data-driven modeling using machine learning techniques can compensate for the limitation of physics-based models. Radke presented a data-driven model that output wildfire spread prediction results [17]. Since the model was trained with actual wildfire data, it showed high prediction accuracy in actual wildfire scenarios. However, the data-driven models have dependency on the training data, meaning that it can result in unreliable spread prediction in untrained conditions of disaster spread. In addition, even when creating a data-driven model for a new situation, the problem of the inability to acquire observation data remains, making it difficult to create a model for general application to various scenarios.

In this study, we propose a digital twin with similarity-based hybrid modeling. The hybrid modeling scheme generates a hybrid disaster behavior model that fuses a physics-based disaster behavior model and a data-driven error correction model to enhance the accuracy of disaster progress prediction. The similarity-based hybrid modeling technique includes a dataset construction procedure by using the similarity assessment between the disasters. This enables the hybrid model to accurately predict the disaster progress even if there are few observation data for the target disaster. In addition, the proposed digital twin includes the execution logic that operates independently of the application system. The application system can use the digital twin service by request through the interface of the digital twin without understanding the logic and technologies for the digital twin.

## 3. The Proposed Digital Twin

This section describes the software architecture of the proposed digital twin. Symbols and descriptions for the proposed digital twin are described in Table 2.

### 3.1. The Proposed Digital Twin Architecture

**Figure 1:**
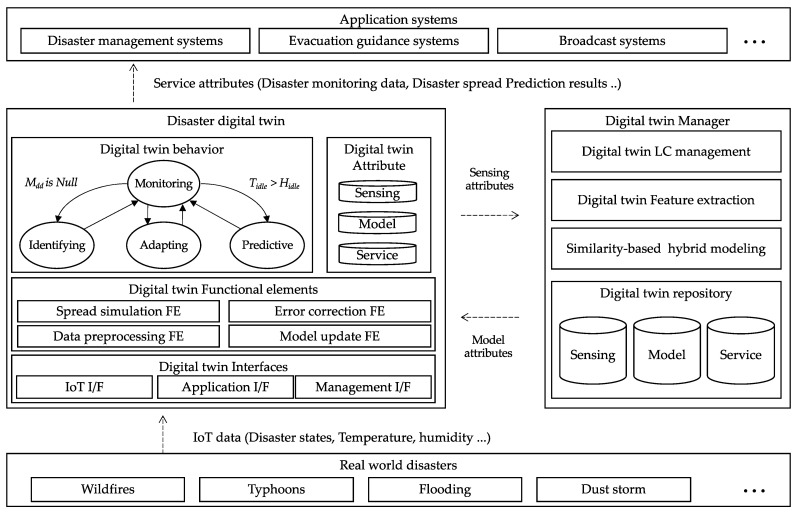
The proposed digital twin architecture.

Figure 1 shows a theoretical software architecture of the hybrid model-based digital twin. The digital twin works with digital twin behaviors (DT-Bs), digital twin functional elements (DT-FEs), and digital twin attributes (DT-As). Digital twin behaviors (DT-B) are operation models of the digital twin. DT-Bs are defined in the form of a state machine to describe the operation procedures of the digital twin. DT-Bs of the proposed digital twin are composed of four behaviors: (1) monitoring behavior to collect observation data from the physical twin, (2) identifying behavior to analyze features of the physical twin, (3) predictive behavior to estimate the future state of the physical twin, and (4) adapting behavior to optimize the digital twin to the physical twin. DT-FEs refer to the computing features of the digital twin to provide digital twin service, such as monitoring, fault detection, and state prediction for the physical twin. For example, physics-based simulation, fault detection, and data preprocessing can be performed in the digital twin. Digital twin attributes (DT-As) are collections of data that represent the state of the digital twin. DT-As include sensor attributes that store observation data from the physical twin, model attributes that store computing model specifications for the digital twin, and service attributes that are the data processed by the digital twin. The digital twin interface consists of an IoT interface that collects observation data from physical twins to acquire sensing attributes, an application interface that exchanges service attributes with an application system, and a management interface that supports interaction with the digital twin manager.

### 3.2. Digital Twin Behavior for the Disaster Digital Twin

The DT-Bs for the disaster digital twin consist of four behaviors: monitoring, identifying, predictive, and adapting.

#### 3.2.1. Monitoring Behavior

**Figure 2:**
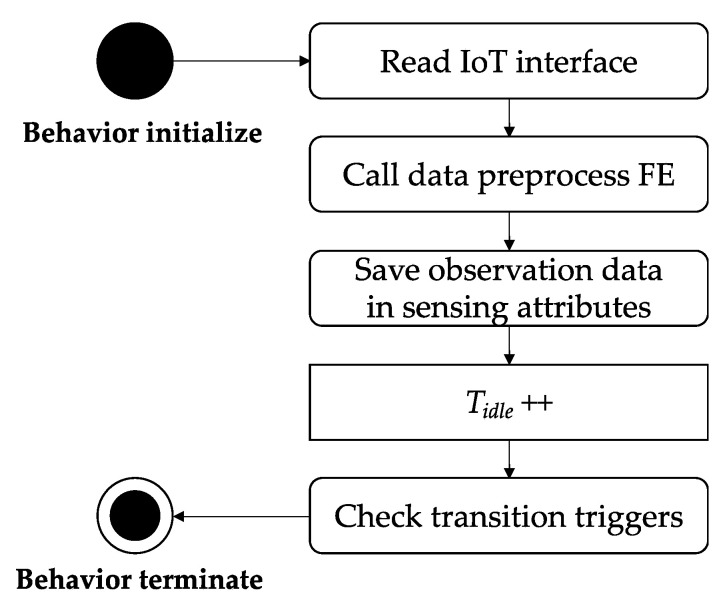
The sequence diagram for monitoring behavior.

For monitoring behavior, the digital twin updates the sensing attributes to track the real-time status of the physical twin by collecting disaster observation data through the IoT interface. Since the collected data are in different formats depending on the type of disaster or the IoT device, preprocessing is required for the digital twin to use it. The digital twin uses data preprocessing FE to process the data. Figure 2 shows a sequence diagram for monitoring behavior.

The data preprocessing FE converts raw IoT data into DT-As so that it can be used in other DT-FEs. Algorithm 1 shows the procedure of the data preprocessing FE. It is executed by inputting the digital twin attribute, its name, and the data shape. The values of the digital twin attribute are filtered according to the basis for each attribute to reduce errors and redundancies. Next, outliers are removed. Then, the shape and data format are converted. If simultaneous processing of multiple digital twin attributes is needed, the above process is repeated for each digital twin attribute.
**Algorithm 1** Data preprocessing functional element**Input**: *Attr*_1_ … *Attr_N_*, *Shape***Output**: *Results*  1: Results ← list()  2: **for**
*i* ← 1 to *N*
**do**  3:  **if** isFiltered(*Attr_i_*) is True **then**  4:      cleansing(*Attr_i_*)  5:      resizing(*Attr_i_*)  6:      formatting(*Attr_i_*)  7:      *Results*.append(*Attr_i_*)  8: **else**  9:      continue 10: **end if** 11: **end for**

#### 3.2.2. Identifying Behavior

**Figure 3:**
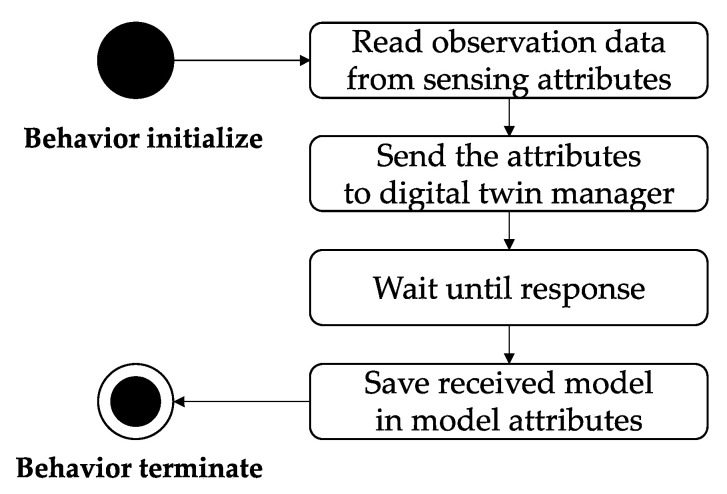
The sequence diagram for identifying behavior.

The digital twin needs a data-driven error correction model optimized to the disaster environments to perform disaster situation prediction using a hybrid model. In the early stage of a disaster, it is difficult to gather the observation data on the disaster to train the optimal data-driven error correction model. The digital twin executes the identifying behavior after the first monitoring behavior to obtain a hybrid prediction model of the current disaster. In identifying behavior, the digital twin transfers the disaster observation data to the digital twin manager to request an optimal hybrid model. Figure 3 is the sequence diagram for the identifying behavior. First, the digital twin selects data that are affected by the disaster, such as weather, climate, terrain, and vegetation data, from the sensing attributes. It transfers the selected data to the digital twin manager. The digital twin waits until the manager returns to a hybrid model. Then, the model is stored in the model attributes and transitions to the monitoring behavior.

#### 3.2.3. Adapting Behavior

**Figure 4:**
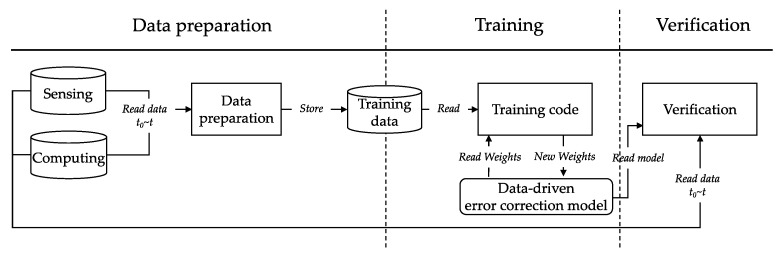
The working procedure of model update FE.

For adapting behavior, the digital twin evaluates and updates the performance of the hybrid model. The digital twin uses the model update FE for the adapting behavior. As the disaster progresses, actual disaster spread data are accumulated in the sensing attributes. The digital twin reads observation data for the disaster progress from the sensing attributes. Then, the digital twin reads the prediction result by the hybrid model stored in the service attribute according to the time of the observation data. The prediction accuracy of the hybrid model is evaluated by comparing the two datasets. If the prediction accuracy is lower than the threshold specified by the digital twin developer, the digital twin uses the model update FE to adapt the model to the current disaster. Figure 4 shows the working procedure of the model update FE.

Model update FE provides a function for the digital twin to improve the performance of the data-driven model through real-data-based continuous learning. The operational pipeline of the model update FE is shown in Figure 4. It is composed of a training dataset by using the I/O data of the hybrid model and IoT acquisition data. The training is performed by inputting the dataset into the data-driven error correction model. After training, the hybrid model is run to check its performance. Even in the process of spreading a disaster through the model update FE, the model is changed to better optimize the target disaster environments.

#### 3.2.4. Predictive Behavior

**Figure 5:**
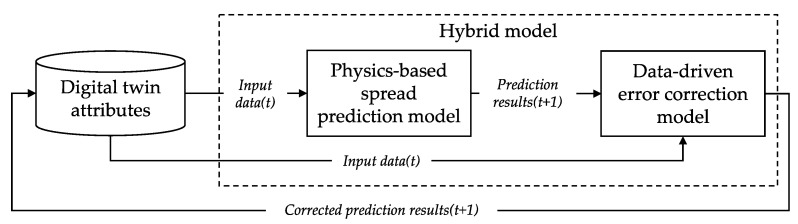
The data flow of the hybrid model for enhanced disaster spread prediction.

For the predictive behavior, the digital twin predicts the disaster progress based on the hybrid model. As shown in Figure 5, the hybrid model is implemented as a series of physics-based spread models and data-driven error correction models. First, the digital twin obtains the input data for the hybrid model from the properties. Prediction results are calculated with the physics-based spread model. Then, the prediction results are processed with the data-driven error correction model to enhance the accuracy of the results. The digital twin uses the disaster simulation FE that predicts disaster progress with the physics-based spread model and the error correction FE that improves the accuracy of the prediction results with the data-driven error correction model.

The disaster simulation FE estimates the disaster progress prediction results with a physics-based spread prediction model. First, the digital twin reads the physics-based spread model from the model attributes and the disaster observation data from the sensing attribute. The disaster simulation FE uses the data to set an initial state value for simulation with the model. Then, the FE results in disaster progress prediction for the simulation time step.

**Figure 6:**
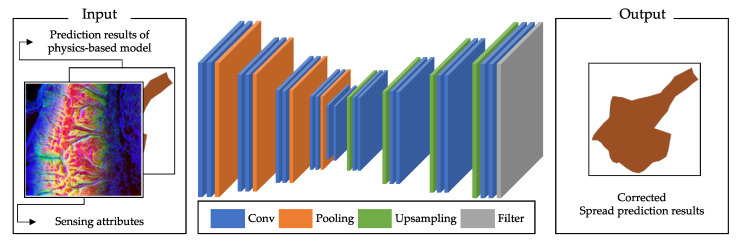
The network structure of the CNN-based model for the data-driven error correction FE.

The data-driven error correction FE provides a function to accurately correct the disaster spread prediction results from the physics-based spread simulation FE. This functional element of the hybrid model-based digital twin was implemented based on the CNN structure-based image-to-image model. The structure of the model is shown in Figure 6. The model receives sensing attribute information used as a simulation input and a prediction result obtained through simulation and outputs a corrected result.

### 3.3. Digital Twin Manager

The digital twin manager consists of a digital twin LC control module that controls the execution and the termination of the digital twin, a feature extraction module that processes DT-As to characterize the digital twin, a similarity-based hybrid modeling module that supports hybrid model training by configuring similarity-based training data, and a digital twin repository that stores the attributes of the existing digital twins.

#### 3.3.1. Digital Twin LC Control Module

The digital twin LC control module controls the digital twin according to the software life cycle from creation, execution, and termination. It configures initial digital twin attributes and digital twin interfaces to ensure that the digital twin can operate. If the digital twin is not updated for a certain period due to the end of the disaster situation, this module terminates the digital twin and returns the DT-As of the digital twin.

#### 3.3.2. Feature Extraction Module

Observational data on the natural environment can be expressed as complex multichannel and multidimensional data spanning various domains, such as vegetation, climate, weather, and topography. This makes the similarity-based hybrid modeling schemes take a lot of time to compare each piece of observation data, and it might be difficult to search for similar disasters through comparison with historical disaster data on time. The existing climate classification and vegetation classification systems only classify macro classifications. This might cause difficulties in finding similar disasters. The feature extraction module uses a machine learning model to extract a unique feature vector of each disaster from the disaster observation data. Disasters with similar characteristics are arranged to have approximate vector values, enabling similarity-based hybrid modeling.

**Figure 7:**
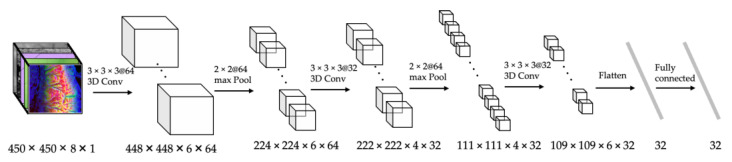
An example of a CNN-based feature extraction for wildfire disaster.

It is developed based on a CNN classification model trained by using disaster observation data as an input and a macro geoscientific classification as a label. An example of the CNN-based feature extraction model for the wildfire disaster is shown in Figure 7. In the training stages, the feature extraction model is trained using a climate classification system as the label, which is a representative classification system related to the major factor in the spread of wildfires, such as vegetation and weather. In the inference phase, it is used after removing the last dense layer of the model. The multidimensional feature vector output from the previous layer of the output layer is used as the feature vector. The vectors extracted from the hidden layer can show more various aspects about the physical system than the classification result.

#### 3.3.3. Similarity-Based Hybrid Modeling Module

**Figure 8:**
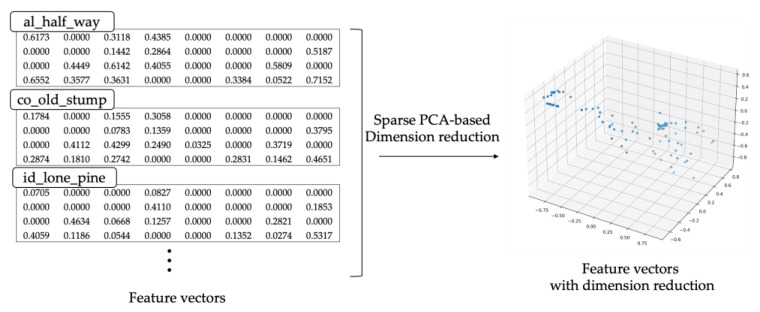
An example of sparse PCA-based dimension reduction in the wildfire dataset.

Similarity-based hybrid modeling modules find similar historical disaster observation data through distance comparison between feature vectors of the disaster observation data and train the hybrid model with the DT-As of the similar digital twin. An example of a similar digital twin search operation based on a dimensionality reduction algorithm for the feature vectors is shown in Figure 8. The feature vectors in Figure 8 represent the climatic characteristics of the input disaster environment data.

In the case of a 32-dimensional vector extracted through the feature extraction FE, it takes a lot of time to calculate the distance between vectors. Thus, it is difficult to set up a model in the early stage. Therefore, distance comparison is performed by reducing it to a 3D vector through a dimension reduction algorithm based on the principal component analysis technique [18]. It orders the existing digital twins based on the distance between feature vectors and selects the N nearest digital twins. The training dataset for hybrid modeling is generated by acquiring observation data from the DT-As of the selected digital twin. After training a hybrid model using the training data, the model is returned to the digital twin. Algorithm 2 shows the algorithm for the proposed hybrid modeling.
**Algorithm 2** Similarity-based hybrid modelingInput: *F_target_*, *F*_1_ … *F_N_*, *N*_1_… *N_N_*, *S*Output: *M_dd_*  1: *Dict_distance_* ← dict()  2: *F_target_* ← dimReduction(*F_target_*)  3: **for**
*i* ← 1 to N **do**  4:  *F_i_* ← dimReduction(*F_i_*)  5:  *Dict_distance_*[*N_i_*] ← calcVectorDistance(*F_target_*, *F_i_*)  6: **end for**  7: sortByValue(*Dict_distance_*)  8: Datatraining←list()  9: *keyList* ←getKeys(*Dict_distance_*) 10: **for** *i* ← 1 to *S* **do** 11:  appendList(*Data_training_*), getAttributes(*keyList*[*i*]) 12: **end for** 13: *M_dd_* ← loadEmptyModel() 14: trainModel(*M_dd_*, *Data_training_*)

## 4. Case Study

The performance of the proposed hybrid model-based digital twin was evaluated in wildfire scenarios, which is a representative natural disaster. Symbols for this section are shown in Table 3.

### 4.1. Simulation Setups

The experiment was performed to evaluate the accuracy and the data independency of the hybrid model and the performance of the prediction models with or without feature similarity-based hybrid modeling. FARSITE, developed based on Rothaermel’s wildfire spread model, was used as a physics-based model for the simulations [15]. The data-driven model was implemented based on the CNN structure that inputs climate data, vegetation data, and the initial wildfire state and outputs the fire spread area. The rest of the CNN model, except for the input layer, was implemented as shown in Figure 6. For the hybrid model, FARSITE was used for the physics-based spread prediction FE, and the CNN-based model presented in Figure 6 was used as the data-based error correction FE.

The accuracy of the proposed digital twin is evaluated through comparison prediction errors of the hybrid model with those of the existing model. The prediction errors of the models are derived in the wildfire scenarios in Table 4. The prediction error is calculated using Equation (1). The prediction error of a wildfire is normalized based on the prediction error of the physics-based model since the scale of the error generated by each model can be different depending on various factors, such as vegetation, climate, and the initial size of the wildfire.

(1) $$E=\frac{1}{x*y}\overset{x}{\underset{i=1}{\sum }}\;\overset{y}{\underset{j=1}{\sum }}Abs\left(P_{i,j}-O_{i,j}\right)$$

The data independency is evaluated by comparing the prediction accuracy of the model on the untrained wildfire data with that of the model on the trained wildfire data. In the training phase of the data-driven model and the hybrid model, it is used as training data after randomly excluding some of the total forest fire data for each model. After the learning is completed, the prediction error increment ratio of the models on the untrained wildfire is calculated. Equation (2) shows the formula for the prediction error increment ratio of the prediction error as follows:

(2) $$I=\frac{Avg\left(E_{ut}\right)-Avg\left(E_{t}\right)}{Avg\left(E_{t}\right)}\;\left(\%\right)$$

The performance of the similarity-based hybrid modeling is evaluated through the amount of error generated by the developed model in the absence of real data of the target wildfire. We evaluate how much the prediction model developed through the proposed hybrid modeling method reduces the error compared with the model developed using randomly selected wildfire among all wildfires. To avoid biased results, the prediction error of the hybrid model with a randomly selected wildfire dataset is averaged after five iterations of the modeling and simulation procedures. Performance evaluation is performed with Equation (3) as follows:

(3) $$R^{wf}=\frac{E_{F}^{wf}-Avg\left(\sum_{i=1}^{5}E_{R\left(i\right)}^{wf}\right)}{Avg\left(\sum_{i=1}^{5}E_{R\left(i\right)}^{wf}\right)}\;\left(\%\right)$$

### 4.2. Simulation Scenarios

The simulation scenarios and the dataset are designed with 63 real-wildfire observation data of wildfires in North America in 2016. Climate data are generated by injecting a random error based on the daily average of each wildfire scenario. Vegetation data are constructed based on the United States Geological Survey LANDFIRE dataset [19]. Wildfire spread data use the Burned Areas Boundaries Dataset of Monitoring Trends in Burn Severity [20]. Table 4 shows the classification of the wildfires used in this simulation according to the Köppen climate classification and the North American wildfire size classification [21,22].

**Figure 9:**
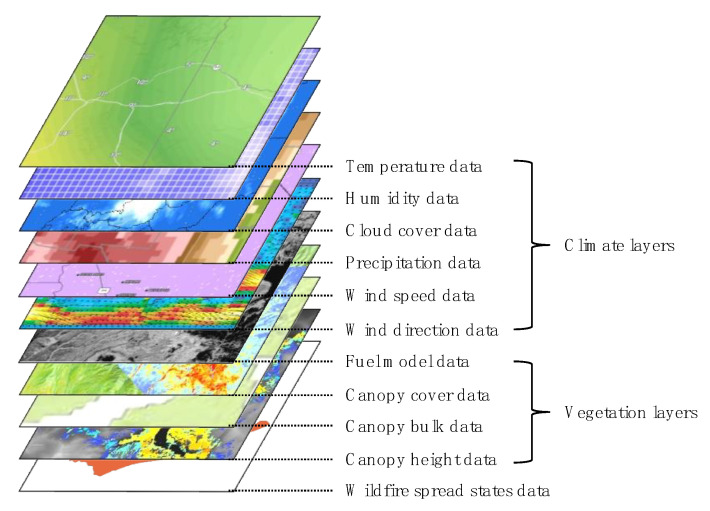
Structure of wildfire dataset for the simulations.

The data for each wildfire scenario consists of 11 channels of 2D data: temperature, humidity, cloud cover, precipitation, wind speed/direction, fuel model, canopy cover/bulk/height and wildfire spread states. The entire data layer is reshaped to the size of 450 × 450. Each data value is normalized as follows: Numerical data which represent as percentile values, including humidity, cloud cover, and canopy cover data, are divided by 100. Wind direction data ranges from 0 to 360 to indicate the direction. They are divided by 360. The rest of the numerical data, including temperature, precipitation, wind speed, canopy bulk, canopy height data, are divided by the maximum for each data type. The fuel model data, which are categorical data, are converted into integer value by sorting them based on the combustibility of each fuel type. A schematic usage data structure is shown in Figure 9. The data-driven model uses all data as input. The data-based error correction FE of the hybrid model uses a vegetation layer, wildfire spread state data, and output results of a physics-based spread prediction FE.

### 4.3. Simulation Results

#### 4.3.1. Simulation Results on the Accuracy of the Hybrid Model

**Figure 10:**
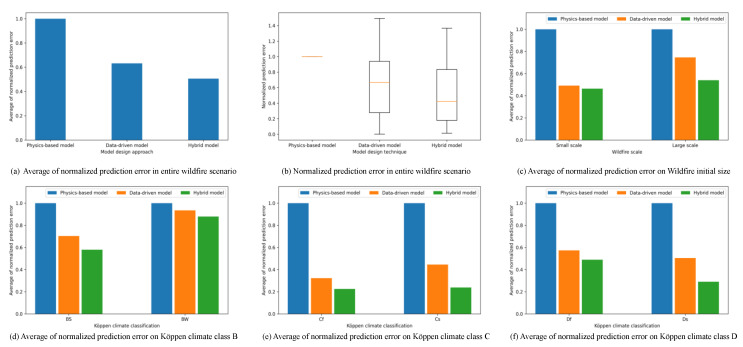
The simulation results on prediction accuracy.

Figure 10 shows the simulation results on the accuracy for the entire wildfire scenario. As shown in Figure 10a, the hybrid model can reduce the prediction error by 40% and 25%, respectively, compared with the physics-based model and the data-driven model in the entire wildfire scenarios. In the case of the physics-based model, it is difficult to calculate the characteristics of each wildfire because it is calculated according to a fixed formula rather than a model optimized for each wildfire. In addition, since the calculation is performed by dividing the actual time into several simulation steps, errors caused by small factors, such as sensor errors can be accumulated, becoming large errors. However, other models show less errors than the physics-based model since they use optimized models based on observation data. The hybrid model has less prediction errors than the data-driven model since the hybrid model uses the formula used by the physics-based model and the spread model learned from the observations at the same time. The formula is used as a base for predictions, the prediction errors decrease, as shown in Figure 10b.

Figure 10c shows the average prediction errors according to the size of initial wildfires. The hybrid model shows the minimum prediction errors in both large and small wildfires. For large wildfires, the models result more prediction errors than for small wildfires because the wildfires can have various wildfire spread patterns and external factors. The hybrid model shows the least prediction error because it compensates the errors from the external factors with the strengths of both models. The hybrid model showed the highest accuracy in the entire climate classification as shown in Figure 10d–f. Since the hybrid model includes the strengths of both the physics-based model and the data-driven model, it has at least 5% less error than the physics-based model, regardless of the climate-specific data ratio in the entire dataset. Therefore, it is confirmed that the hybrid model predicts the disaster spread states with higher accuracy than other models.

#### 4.3.2. Simulation Results on the Data Independency of the Hybrid Model

**Figure 11:**
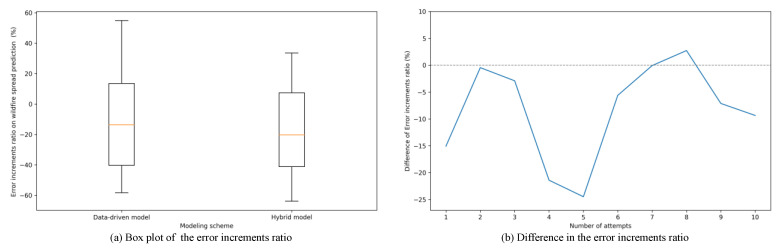
The simulation results on error increments ratio on wildfire spread prediction for untrained wildfire scenarios compared with wildfire spread prediction for trained wildfire scenarios.

Figure 11 shows the results of the data independency by repeating the process of randomly selecting and evaluating a forest fire to be used for learning among all wildfire scenarios ten times. The variation in the error value of the prediction result for the unlearned forest fire compared with the predicted result for the learned forest fire are shown in Figure 11a. The amount of error that occurs in predictions for untrained wildfires is smaller in the hybrid model than in the data-driven model. In the case of a data-driven model, the rate of the error reduction is large, and the rate of the reduction is wide and distributed. Since the data-driven models predict the disaster status only using rules extracted from the dataset, the performance of the model depends on the dataset. On the other hand, the hybrid model shows fewer errors in the increasement ratio than the data-driven model because the hybrid model uses the results of the physics-based prediction model as a reference value. Therefore, the prediction of the hybrid model has less dependency to dataset than that of data-driven model.

#### 4.3.3. Simulation Results on the Proposed Hybrid Modeling Schemes

**Figure 12:**
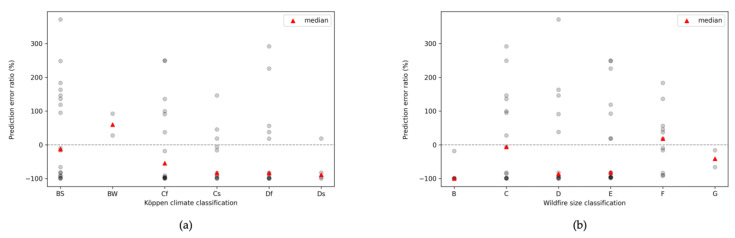
The simulation results on the evaluation on the performance of the proposed hybrid modeling scheme. (**a**) Prediction error ratio for a hybrid model trained with a feature-based selected training data compared a hybrid model trained with a randomly selected training data on Köppen climates class, (**b**) Prediction error ratio for a hybrid model trained with a feature-based selected training data compared a hybrid model trained with a randomly selected training data on initial wildfire size class.

Figure 12 shows the rate of change for the prediction error of the hybrid modeling technique using randomly selected wildfire training data and the proposed similarity-based hybrid modeling technique. Each point in Figure 12 is the evaluation result for each wildfire presented in Table 4. It is confirmed that the proposed modeling scheme makes the model more reliable than the randomly selected data-based hybrid modeling scheme. Since the feature vector used as the basis of the similarity analysis is extracted based on the climate class, errors are reduced where there are multiple wildfires with the same climate classification. Weather and vegetation, which are major factors in the spread of wildfires, are affected by precipitation. In the case of the Df class, although there are few wildfires with the same classification, errors are reduced because there are many Cfs with the same precipitation classification. This means that a hybrid model with good performance can be developed even in a situation where observation data are insufficient by extracting features that well-reflect factors affecting the spread of the disasters and performing hybrid modeling using them. Therefore, the more specifically the characteristics of the disaster are classified, the more that a similar disaster can be found among the existing data. Therefore, it is expected that a hybrid model with good performance for the target disaster can be developed through the proposed similarity-based hybrid modeling technique even if the observation data of the target disaster is insufficient. Additionally, as the amount of existing disaster observation data increases, it is possible to make the hybrid model more reliable by acquiring many observation data from disasters with similar features.

## 5. Conclusions

This study proposes a digital twin architecture with the similarity-based hybrid modeling for dependable disaster management systems. First, a digital twin software structure that operates with a hybrid disaster model is proposed. The digital twin collects observation data and uses it to design and execute the hybrid model. This makes it possible for the digital twin to predict the spread of disasters more reliably than existing techniques. Second, the proposed digital twin architecture can develop an accurate hybrid disaster model by means of similarity-based hybrid modeling schemes. The scheme analyzes the observation data to construct a well-refined training dataset from similar historical disaster data. By training the hybrid models with the dataset, it results small prediction errors despite of insufficient observation data on the corresponding disaster environments. Finally, the continuous learning pipeline is able to keep enhancing the prediction accuracy of the hybrid model based on the real-time data collected as the disaster progresses.

The simulation results with real wildfire scenarios show that the hybrid model can reduce errors by up to half in the disaster spread prediction results compared with the existing disaster models. In addition, it was confirmed that the hybrid model is less affected by the training data compared with the data-based model. Thus, it can show better performance when the model is trained based on the similarity even in a situation where there is insufficient data. Therefore, using the proposed digital twin, it would be possible to more accurately predict the situation of a physical system that can cause a fatal problem, such as a disaster. In future works, the proposed hybrid modeling scheme should be applied to industrial domains. Since types of observation data are diverse in the industrial domains, a multifunctional deep learning-based feature extraction scheme can utilize artificial intelligence models of various structures.

## Figures and Tables

**Table 1:** Comparison of disaster prediction modeling approaches.

Features	Physics-Based Model	Data-Driven Model	Hybrid Model	Proposed Hybrid Model
Accuracy	Low	High in trained scenario	High	High
Data independency	O	X	**△**	O
Adaptability	X	O	O	O

O: Has the feature. **△**: Weakly has the feature. X: Doesn’t have the feature.

**Table 2:** Symbols and description for hybrid model-based digital twin.

Symbol	Description
$M_{dd}$	Model for data-driven error correction FE
$T_{idle}$	Idle time of hybrid model-based digital twin
$H_{idle}$	Threshold of idle time of hybrid model-based digital twin
$T_{sim}$	Simulation time of hybrid model-based digital twin
$T_{end}$	Simulation end time of hybrid model-based digital twin
$T_{simste}$	Simulation time step size of hybrid model-based digital twin
*Attr*	Digital twin attributes
*F*	Feature vector of digital twin
*N*	Name of digital twin
*S*	Size of training data for hybrid modeling

**Table 3:** The symbols and description for the case study.

Symbol	Description
$E_{p}^{wf}$	Error amounts of type of model on disaster name
x, y	The width and height of the data
$P_{i,j}$	Prediction result for coordination *i*,*j*
$O_{i,j}$	Observation data for coordination *i*,*j*
I	Prediction error increments ratio on the untrained data
$E_{t}$	Prediction error on trained wildfires
$E_{ut}$	Prediction error on untrained wildfires
$E_{R\left(i\right)}^{wf}$	Prediction error with randomly selected wildfire dataset
$E_{F}^{wf}$	Prediction error with similarity-based hybrid modeling
$R^{wf}$	Prediction error ratio for *wf* wildfire

**Table 4:** Real data-based wildfire scenarios by Köppen climate classification and size class classification.

Size Class		Köppen Climate Classification					
		BS	BW	Cf	Cs	Df	Ds
Small	B	az_sunflower	-	ga_chimney_top al_caney_head	or_mr_068_blue_top	co_silver_creek	id_gleason
	C	az_jack co_long_draw az_juniper az_pivot_rock	az_skeleton	al_half_way al_lookout_mountain ga_burrell_42 ga_creek_road ar_whitaker_point	or_gold_canyon ca_ash	co_old_stump co_rosebud	id_freeman
	D	az_maple az_fresnal az_fuller az_airstrip		ga_burrell ga_irwin_mill	-	id_moose co_freeman id_comet co_starwood id_black	-
Large	E	ca_cedar_sqf co_happy_hollow az_choulic az_mormon	az_tenderfoot	fl_taylor ga_tatum_gulf al_power_horn ga_rocky_face ga_rock_mountain	or_draw id_pioneer id_john_doe	co_spring_creek_2 co_cold_springs	id_dry_creek id_buck
	F	az_brown az_cowboy nv_horseshoe	-	nv_pinto ga_fox_mountain_fire	nv_little_valley or_rail ca_chimney_cnd	id_lone_pine co_hayden_pass	-
	G	nv_maggie	-	-	or_rattlesnake	-	-

## Data Availability

Not applicable.
